# Associations of Solid Fuel Use and Circadian Rhythm Syndrome With Physical Function and Muscle Strength in Middle-Aged and Older Adults: Nationwide Cohort Study in China

**DOI:** 10.2196/78352

**Published:** 2026-06-29

**Authors:** Haochen Wang, Pengsen Mou, Donghao Liu, Ze Zhao, Wenzheng Chen, Yuxin Yao, Haoyan Shi, Jiaxin Guan, Jing Dong, Yingliang Wei

**Affiliations:** 1 Department of Orthopaedics Shengjing Hospital China Medical University Shenyang, Liaoning China; 2 Department of Orthopedics Union Hospital, Tongji Medical College Huazhong University of Science and Technology Wuhan, Hubei China; 3 Key Laboratory of Environmental Stress and Chronic Disease Control and Prevention, Ministry of Education China Medical University Shenyang, Liaoning China; 4 Department of Occupational and Environmental Health School of Public Health China Medical University Shenyang, Liaoning China

**Keywords:** age-related health decline, circadian rhythm syndrome, household air pollution, muscle strength, physical function

## Abstract

**Background:**

The relationships among circadian rhythm syndrome, physical function, and muscle strength remain unclear.

**Objective:**

This study aimed to demonstrate the separate and combined deleterious effects of solid fuel use and circadian rhythm syndrome on physical function and muscle strength.

**Methods:**

We used data from the China Health and Retirement Longitudinal Study cohort. The study population consisted of participants who underwent comprehensive assessments of metabolism, circadian rhythm, indoor air pollution, physical function, and muscle strength at the initial evaluation. Muscle strength was assessed using repeated grip strength measurements, and physical function was assessed using a composite score of muscle strength, physical performance, and balance. Circadian rhythm syndrome was derived from the 5 diagnostic components of metabolic syndrome combined with sleep duration and depression. Logistic regression and linear mixed models were used to assess the relationships among solid fuel use, circadian rhythm syndrome, physical function, and muscle strength. Furthermore, we analyzed the mediating role of circadian rhythm syndrome and its combined effect with solid fuel use on physical function and muscle strength.

**Results:**

A total of 7934 participants were included in the study, most of whom used solid fuels. Solid fuel use was positively associated with circadian rhythm syndrome (odds ratio [OR] 1.078, 95% CI 1.031-1.125; *P*<.05). Circadian rhythm syndrome was found to be a significant risk factor for impairment of physical function (*β*=−0.475; *P*<.05) and muscle strength (*β*=−0.304; *P*<.05). Participants who used solid fuels and had circadian rhythm syndrome needed to pay more attention to changes in physical function (*β*=−0.698; *P*<.05) and muscle strength (*β*=−0.332; *P*<.05). A significant interaction was observed between solid fuel use and circadian rhythm syndrome on physical function (*P*_interaction_=.03) and muscle strength (*P*_interaction_=.02). Circadian rhythm syndrome partially mediated the association between solid fuel use and physical function, accounting for 2.51% of the total effect.

**Conclusions:**

Circadian rhythm syndrome exacerbates the adverse effects of solid fuel use on physical function and muscle strength. Fuel cleanliness and regular work and rest habits are crucial for the health of middle-aged and older adults.

## Introduction

Physical function assesses the ability to perform basic behavioral activities in terms of muscle strength, upper and lower extremity function, and balance [1]. Maintaining adequate physical function preserves the ability to live independently, optimizes quality of life, improves social participation, and reduces the risk of disability [2]. Low muscle strength and reduced physical function are associated with frailty, an increased risk of falls and fractures, and consequently, high morbidity and mortality [3-5]. Given that there are currently no effective pharmacological treatments to delay age-related decline in physical function [6], it is important to explore the risk factors associated with declining physical function and identify high-risk populations that are more likely to benefit from interventions to mitigate the decline in physical function.

The circadian rhythm is a biological rhythm with a cycle of approximately 24 hours that adapts to environmental changes and maintains stable physiological functions [7]. The suprachiasmatic nucleus of the hypothalamus is the central pacemaker of circadian rhythms and maintains synchronization with the external environment through neurohumoral signals in coordination with molecular oscillators in peripheral tissues [8]. Circadian rhythm is highly tissue-specific; each tissue of the human body, including the musculoskeletal system, has its own unique biological clock, which maintains physical function to meet daily demands by regulating the transcription and translation of clock genes [9]. The robustness of circadian rhythms decreases with age and the disruption of circadian rhythms; this is known as circadian rhythm syndrome and is a mechanism that potentially leads to an increased risk of various diseases [10,11]. Although maintaining a normal circadian rhythm is critical for skeletal muscle metabolic homeostasis, muscle fiber number and volume, and joint health [12], the impact of circadian rhythm syndrome on physical function and muscle strength lacks high-quality epidemiological studies for confirmation. As such, it is necessary to elucidate the relationship between circadian rhythm syndrome, physical function, and muscle strength and to identify special populations susceptible to circadian rhythm syndrome.

Household air pollution from solid fuel use is a common environmental safety problem worldwide [13]. According to statistics, the use of solid fuels is more common in developing countries. In China, approximately 450 million people rely heavily on solid fuels [14]. Numerous studies have shown that solid fuel use significantly increases the risk of physical and mental illnesses, such as cardiovascular disease and depression [14,15]. Additionally, our previous research also found adverse effects of solid fuel use and a combined adverse effect of solid fuel use and airborne particulate matter on physical function [1]. However, the association between solid fuel use and circadian rhythm syndrome remains unclear. Furthermore, solid fuel use and circadian rhythm syndrome are both risk factors for aging [16], and their combined effects on physical function and muscle strength require further elucidation.

To address these gaps, we performed a nationwide population-based cohort study to assess the independent and combined associations of solid fuel use and circadian rhythm syndrome with physical function and muscle strength in middle-aged and older adults. This study further aimed to explore the potential role of circadian rhythm syndrome in the relationship between solid fuel use and functional outcomes. The findings are expected to provide evidence relevant to public health strategies targeting environmental and lifestyle risk factors in aging populations.

## Methods

### Study Design and Data Source

This study used data from the China Health and Retirement Longitudinal Study (CHARLS), a large nationally representative cohort study of middle-aged and older adults in China [17]. CHARLS randomly selected 150 counties from 28 provinces in China, with a total of 17,708 participants as baseline and followed up every 2 years using face-to-face computer-assisted personal interviews (wave 2 in 2013, wave 3 in 2015, wave 4 in 2018, and wave 5 in 2020).

In the CHARLS survey, comprehensive assessments of circadian rhythm syndrome, physical function, and muscle strength were available only in the 2011, 2013, and 2015 waves. Therefore, data from 17,708 participants in 2011 (wave 1) were used as the baseline, with longitudinal analyses conducted using data from 2015 (wave 3). Exclusion criteria were: (1) being aged younger than 45 years (n=321); (2) no information on circadian rhythm syndrome (n=6067), physical function (n=2712), or household fuel use (n=674); and (3) no follow-up data (n=2916), comprising participants with missing information on circadian rhythm syndrome (n=929), physical function (n=1681), and household fuel use (n=306). A total of 7934 participants were finally included in the cross-sectional study, and 5018 participants in the longitudinal analysis (Multimedia Appendix 1). As shown in Multimedia Appendix 2, all participants in this study were distributed across 126 cities in 28 provinces in China, with the majority located in southern, central, and western China.

### Household Solid Fuel Use Measurement

Household solid fuel use was assessed using a structured questionnaire. Participants were considered to use solid fuels when they answered “coal,” “crop residue/wood burning,” or “other” for heating and/or cooking, and clean fuels were considered to be used when they answered “natural gas,” “marsh gas,” “liquefied petroleum gas,” “solar,” or “electricity” for heating and/or cooking. Of note, due to the lack of data on the type of heating fuel used during follow-up, we conducted a longitudinal analysis of cooking fuels only and used participants using clean fuels as a reference to assess the impact of solid fuel use on circadian rhythm syndrome, physical function, and muscle strength. For the cross-sectional analysis, since data on both cooking and heating fuels were available, we adopted a 3-category classification to comprehensively reflect household fuel use patterns: clean fuel (reference group), partial solid fuel use (solid fuel used for at least 1 of cooking or heating), and solid fuel use.

### Circadian Rhythm Syndrome Assessment

Based on previous studies, we used circadian rhythm syndrome as assessed by a combination of the 5 variables of the International Diabetes Federation assessing metabolic syndrome (elevated blood pressure, blood glucose, triglycerides, waist circumference, and lowered high-density lipoprotein cholesterol), combined with sleep duration and depression [11,18]. Detailed diagnostic criteria are shown in Multimedia Appendix 3 [19,20]. Participants were diagnosed with circadian rhythm syndrome if they met 4 of the 7 components [11,18].

### Physical Function Assessment

Grip strength is a validated proxy for overall skeletal muscle strength and is widely used for sarcopenia screening in middle-aged and older adults [21-23]; therefore, it was used to assess muscle strength in this study. Each participant held the TM WL-1000 dynamometer (Nantong Yuejian Physical Measurement Equipment Co Ltd) in both hands successively at full strength for 2 separate measurements. The maximum of the 4 recordings was used and normalized by body weight [24]. If the participant was unable to perform the test with 1 hand due to special reasons, the maximum value of the other hand was recorded. In addition, the physical performance of each participant was assessed using gait speed and the chair stand test [23]. The above 3 tests were assigned a score of 1 to 4 to the participant based on the results from the first quartile to the fourth quartile. If the participant was unable to complete the test, the test was assigned a score of 0. Physical function was assessed using the above 3 tests plus the balance test [1]. For the balance test, participants were asked to stand and hold for 10 seconds in 3 increasingly difficult positions with their feet side-by-side, in semi-tandem, and in full-tandem. Scores were assigned based on the results of the tests (0: side-by-side <10 seconds; 1: side-by-side ≥10 seconds but semi-tandem <10 seconds; 2: semi-tandem ≥10 seconds and full-tandem <3 seconds; 3: semi-tandem ≥10 seconds and full-tandem between 3 and 10 seconds; 4: semi-tandem ≥10 seconds and full-tandem ≥10 seconds). Based on these assessments, the total physical function score was calculated as the sum of 4 domains: muscle strength, gait speed, chair-stand performance, and balance. Muscle strength, gait speed, and chair-stand test were each scored from 0 to 4 according to quartile-based performance, while balance was scored from 0 to 4 based on predefined criteria. Accordingly, the composite physical function score ranged from 0 to 16, with higher scores indicating better overall physical function [25-27].

### Covariates

The following basic characteristics were included in the main analysis [1,11,18]: age, gender, education level, marital status, urbanization level, smoking status, drinking status, annual income, indoor temperature, housing type, BMI, and a comprehensive set of chronic diseases. Detailed information is provided in Multimedia Appendix 4.

### Statistical Analysis

#### Statistical Summary of Participant Characteristics

The baseline characteristics of the participants were summarized based on the types of cooking and heating fuels used. Continuous variables are presented as mean (SD), while categorical variables are expressed as frequencies (percentage). Differences between groups were assessed using 2-tailed *t* tests for continuous variables and chi-square tests for categorical variables.

#### Cross-Sectional and Longitudinal Analysis of Separate Effects

We examined the relationships among solid fuel use, circadian rhythm syndrome, and physical function scores in both cross-sectional and longitudinal studies. Detailed information is provided in Multimedia Appendix 4. The missing rate for most individual characteristics was <5%; thus, missing values were imputed using a predictive mean-matching method to avoid excessive sample loss [18,28]. Upon comparing the summary statistics of the imputed dataset with those of the original dataset, no significant differences in the basic characteristics were observed (Multimedia Appendix 5). All statistical analyses were performed using R (version 4.3.1; R Foundation for Statistical Computing). Two-sided tests with a *P* value <.05 were considered significant.

#### Combined Effects and Interaction Analysis

We created a new variable by combining fuel type and circadian rhythm syndrome, resulting in 4 categories (2 × 2): fuel type (clean or solid fuels) and circadian rhythm syndrome (yes or no), to explore the collective effects of the 2 exposures on physical function scores. Moreover, we added multiplicative interaction terms to the model to analyze the interaction effect of solid fuel use and circadian rhythm syndrome on physical function scores. A *P* value <.05 for the interaction indicated the presence of a multiplicative interaction.

#### Stratified Analysis

Concurrently, we stratified by fuel type and circadian rhythm syndrome to explore the impact of other exposures on physical function scores, as well as the scores of the 4 tests.

#### Mediation Analysis

Finally, we conducted a mediation analysis using the R package *mediation* to assess whether circadian rhythm syndrome mediates the association between solid fuel use and physical function scores. Detailed information and sensitivity analyses are provided in Multimedia Appendix 4.

### Ethical Considerations

Ethical approval was obtained from the Ethics Review Committee of Peking University (approval number IRB00001052-11015), and all participants consented to participate in this study.

## Results

### Statistical Summary of Participant Characteristics

Table 1 shows the baseline characteristics of the 7934 participants enrolled in the study. The findings revealed that solid fuel was the predominant household fuel, with 4820 (60.8%) and 4827 (60.8%) participants using it for cooking and heating, respectively. Participants who used solid fuels were generally older (aged 59.6, SD 8.9 years), female (2522/4820, 52.3%), less educated (4563/4820, 94.7%), living in rural areas (3827/4820, 79.4%), married (4078/4820, 84.6%), never smokers (3240/4820, 67.2%), never alcohol drinkers (3231/4820, 67%), with lower annual household incomes (3372/4820, 70%), and living in low-rise buildings (3639/4820, 75.5%) with unsuitable indoor temperatures. In terms of health status, participants who used solid fuels were more likely to have various chronic conditions, had a higher prevalence of circadian rhythm syndrome (1923/4820, 39.8%), and had lower physical function (12, SD 2.4), muscle strength (2.5, SD 1.1), gait speed (3.3, SD 1.1), chair stand test (2.4, SD 1.1), and balance (3.9, SD 0.5) scores. Characteristics of the participants in the longitudinal analysis are presented in Multimedia Appendix 6. Multimedia Appendix 7 illustrates the national distribution of solid fuel use, prevalence of circadian rhythm syndrome, physical function, and muscle strength. Northern China exhibited a higher prevalence of circadian rhythm syndrome and solid fuel dependency than Southern China, whereas physical function and grip strength scores were lower in the North.

**Table 1:** Baseline characteristics of the study participants according to household fuel types. Continuous and categorical variables were compared using analyses of independent sample Student 2-tailed *t* test and chi-square tests, respectively.

Variable				Cooking fuels				*P* value		Heating fuels				*P* value
				Clean (n=3114)		Solid (n=4820)				Clean (n=3107)		Solid (n=4827)		
Demographic characteristics														
	Age (years), mean (SD)		57.3 (8.8)		59.6 (8.9)		<.001		58.5 (9.1)		58.9 (8.8)		.04	
	Gender, n (%)						.99						.71	
		Male	1484 (47.7)		2298 (47.7)				1473 (47.4)		2309 (47.8)			
		Female	1630 (52.3)		2522 (52.3)				1634 (52.6)		2518 (52.2)			
	Education level, n (%)						<.001						<.001	
		Middle school or below	2723 (87.4)		4563 (94.7)				2783 (89.6)		4503 (93.3)			
		High or vocational school	349 (11.2)		240 (5)				282 (9.1)		307 (6.4)			
		Above high school	42 (1.3)		17 (0.4)				42 (1.4)		17 (0.4)			
	Residence, n (%)						<.001						<.001	
		Urban	1529 (49.1)		993 (20.6)				1376 (44.3)		1146 (23.7)			
		Rural	1585 (50.9)		3827 (79.4)				1731 (55.7)		3681 (76.3)			
	Marital status, n (%)						.14						.02	
		Married and living	2607 (83.7)		4078 (84.6)				2583 (83.1)		4102 (85)			
		Married but separated	175 (5.6)		223 (4.6)				181 (5.8)		217 (4.5)			
		Single or other	332 (10.7)		519 (10.8)				343 (11)		508 (10.5)			
	Smoking status, n (%)						.005						.001	
		Never	2187 (70.2)		3240 (67.2)				2191 (70.5)		3236 (67)			
		Current	927 (29.8)		1580 (32.8)				916 (29.5)		1591 (33)			
	Alcohol drinking status, n (%)						.38						.63	
		Never	2058 (66.1)		3231 (67)				2081 (67)		3208 (66.5)			
		Current	1056 (33.9)		1589 (33)				1026 (33)		1619 (33.5)			
Household characteristics, n (%)														
	Housing type						<.001						<.001	
		One-story	1728 (55.5)		3639 (75.5)				1546 (49.8)		3821 (79.2)			
		Multistory	1386 (44.5)		1181 (24.5)				1561 (50.2)		1006 (20.8)			
	Indoor temperature						<.001						<.001	
		Bearable	2716 (87.2)		3943 (81.8)				2702 (87)		3957 (82)			
		Hot	320 (10.3)		684 (14.2)				344 (11.1)		660 (13.7)			
		Cold	78 (2.5)		193 (4)				61 (2)		210 (4.4)			
	Annual income, RMB^a^						<.001						<.001	
		≤20,000	1523 (48.9)		3372 (70)				1633 (52.6)		3262 (67.6)			
		>20,000	1591 (51.1)		1448 (30)				1474 (47.4)		1565 (32.4)			
Health status														
	BMI, mean (SD), kg/m^2^		24 (8.2)		23.4 (13.4)		.03		23.8 (11.7)		23.5 (11.6)		.24	
	Cancer, n (%)		28 (0.9)		38 (0.8)		.60		26 (0.8)		40 (0.8)		.97	
	Chronic lung disease, n (%)		263 (8.4)		541 (11.2)		<.001		283 (9.1)		521 (10.8)		.02	
	Heart problem, n (%)		336 (10.8)		543 (11.3)		.51		273 (8.8)		606 (12.6)		<.001	
	Stroke, n (%)		61 (2)		543 (2.3)		.25		56 (1.8)		118 (2.4)		.06	
	Psychiatric problems, n (%)		29 (0.9)		63 (1.3)		.13		28 (0.9)		64 (1.3)		.08	
	Arthritis, n (%)		1021 (32.8)		1766 (36.6)		<.001		1025 (33)		1762 (36.5)		.001	
	Liver disease, n (%)		98 (3.1)		167 (3.5)		.44		104 (3.3)		161 (3.3)		>.99	
	Kidney disease, n (%)		161 (5.2)		309 (6.4)		.02		177 (5.7)		293 (6.1)		.49	
	Digestive disease, n (%)		670 (21.5)		1186 (24.6)		.001		731 (23.5)		1125 (23.3)		.82	
	Asthma, n (%)		115 (3.7)		260 (5.4)		<.001		120 (3.9)		255 (5.3)		.004	
	Memory disorder, n (%)		30 (1)		69 (1.4)		.07		32 (1)		67 (1.4)		.16	
	Circadian rhythm syndrome, n (%)		1181 (37.9)		1894 (39.3)		.22		1152 (37.1)		1923 (39.8)		.01	
Circadian rhythm syndrome component, n (%)														
	Reduced HDL-C^b^		1278 (41)		1839 (38.2)		.01		1200 (38.6)		1917 (39.7)		.33	
	Elevated waist circumference		1922 (61.7)		2561 (53.1)		<.001		1815 (58.4)		2668 (55.3)		.006	
	Hyperglycemia		1697 (54.5)		2837 (58.9)		<.001		1735 (55.8)		2799 (58)		.06	
	Raised blood pressure		1854 (59.5)		2830 (58.7)		.47		1809 (58.2)		2875 (59.6)		.24	
	Elevated triglyceride		940 (30.2)		1291 (26.8)		.001		870 (28)		1361 (28.2)		.85	
	Short sleep duration		861 (27.6)		1529 (31.7)		<.001		920 (29.6)		1470 (30.5)		.42	
	Depressive symptoms		921 (29.6)		2084 (43.2)		<.001		964 (31)		2041 (42.3)		<.001	
Physical function, mean (SD)														
	Physical function score		12.5 (2.4)		12 (2.5)		<.001		12.5 (2.4)		12 (2.4)		<.001	
	Muscle strength score		2.5 (1.1)		2.5 (1.1)		.24		2.5 (1.1)		2.5 (1.1)		.021	
	Gait speed score		3.5 (1)		3.2 (1.1)		<.001		3.4 (1)		3.3 (1.1)		.009	
	Chair stand test score		2.7 (1.1)		2.4 (1.1)		<.001		2.7 (1.1)		2.4 (1.1)		<.001	
	Balance score		3.9 (0.4)		3.9 (0.5)		<.001		3.9 (0.5)		3.9 (0.5)		.69	

^a^RMB 1=US $0.15 as of June 10, 2026.

^b^HDL-C: high-density lipoprotein cholesterol.

### Cross-Sectional and Longitudinal Analysis of Separate Effects

**Figure 1:**
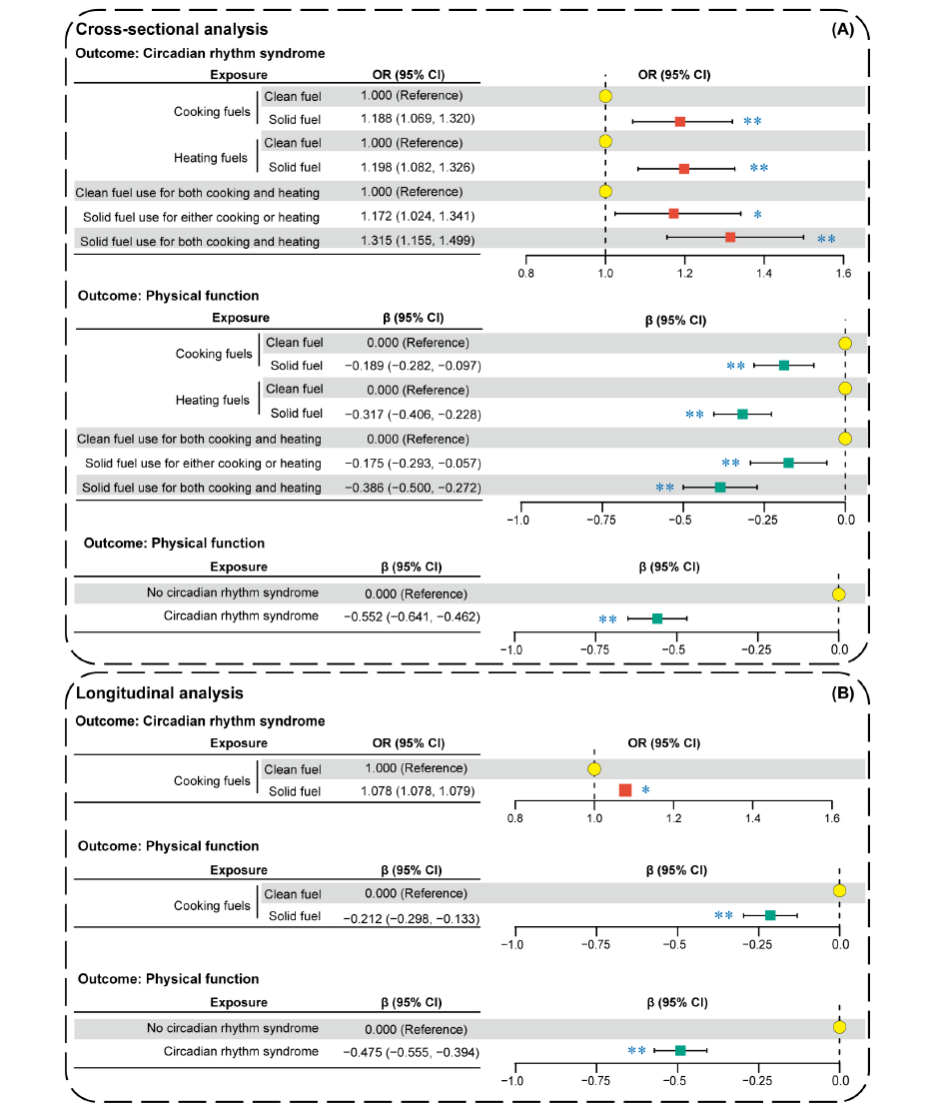
Associations among solid fuel use, circadian rhythm syndrome, and physical function in (A) cross-sectional and (B) longitudinal analyses. Estimates were derived from multivariable regression models (binary logistic regression for circadian rhythm syndrome, linear models for cross-sectional analyses, and mixed-effects linear models for longitudinal analyses). Results shown are from model 4, adjusted for age, gender, BMI, urbanization, education level, marital status, smoking status, drinking status, housing type, indoor temperature, and chronic diseases. A single asterisk (*) indicates *P*<.05 and double asterisks (**) indicate *P*<.01. OR: odds ratio.

Figure 1A displays the separate effects of solid fuel use, circadian rhythm syndrome, and physical function at baseline. Regarding solid fuel use and circadian rhythm syndrome, participants who used solid fuels for cooking and those who used solid fuels for heating exhibited an elevated risk of circadian rhythm syndrome by 18.8% (odds ratio [OR] 1.188, 95% CI 1.069 to 1.32) and 19.8% (OR 1.198, 95% CI 1.082 to 1.326), respectively. Compared with participants using clean fuels for cooking and heating, those partially or entirely reliant on solid fuels experienced an increased risk of circadian rhythm syndrome by 17.2% (OR 1.172, 95% CI 1.024 to 1.341) and 31.5% (OR 1.315, 95% CI 1.155 to 1.499), respectively. Additionally, we examined the influence of solid fuel use on physical function and observed a negative correlation between the use of solid fuels for cooking (*β*=–0.189, 95% CI –0.282 to –0.097) and heating (*β*=–0.317, 95% CI –0.406 to –0.228) and physical function compared with clean fuels. Partial and complete reliance on solid fuels led to reductions in physical function scores by 0.175 (95% CI –0.293 to –0.057) and 0.386 (95% CI –0.5 to –0.272) points, respectively. Further analysis revealed significant decreases in muscle strength (*β*=–0.061, 95% CI –0.117 to –0.005) and chair stand test scores (*β*=–0.273, 95% CI –0.335 to –0.211) among participants who used solid fuels for cooking and heating (Multimedia Appendix 8). After adjusting for all covariates, we found a negative correlation between circadian rhythm syndrome and physical function (*β*=–0.552, 95% CI –0.641 to –0.462). Patients with circadian rhythm syndrome experienced decreased muscle strength, balance, and chair stand test scores (*β=*–0.378, 95% CI –0.421 to –0.335; *β=*–0.032, 95% CI –0.053 to –0.01; and *β=*–0.123, 95% CI –0.172 to –0.073, respectively; Multimedia Appendix 8).

Consistent with the results of the cross-sectional analysis, as shown in Figure 1B, our longitudinal analyses revealed a positive correlation between solid fuel use and the risk of circadian rhythm syndrome (OR 1.078, 95% CI 1.031 to 1.125). Additionally, participants who used solid fuel (*β*=–0.212, 95% CI –0.298 to –0.133) and had circadian rhythm syndrome (*β*=–0.475, 95% CI –0.555 to –0.394) had notably lower physical function scores. Multimedia Appendix 9 further demonstrates that these participants also had notably lower chair stand test and balance scores. Although no correlation was detected in the analyses examining the impact of solid fuel use on muscle strength, lower muscle strength scores were observed in participants with circadian rhythm syndrome (*β*=–0.304, 95% CI –0.342 to –0.265).

### Combined Effects and Interaction Analysis

**Figure 2:**
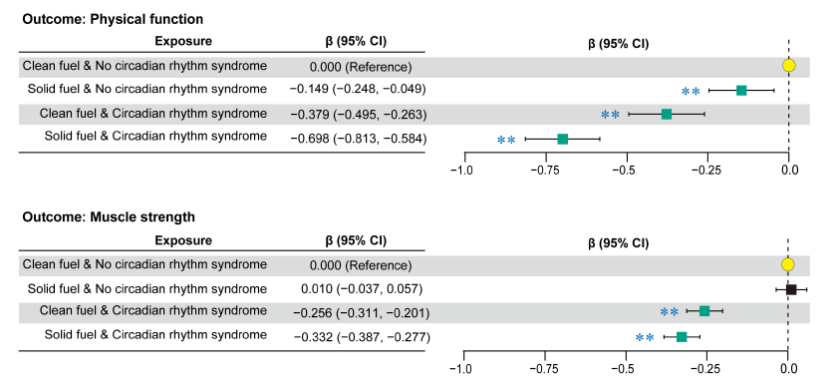
Combined effects of solid fuel use and circadian rhythm syndrome on physical function and muscle strength. Estimates were derived from multivariable linear regression models including a 2 × 2 combined exposure variable (fuel type × circadian rhythm syndrome) and a multiplicative interaction term to assess interaction effects. Adjusted for age, gender, BMI, urbanization, education level, marital status, smoking status, drinking status, housing type, indoor temperature, and chronic diseases. A single asterisk (*) indicates *P*<.05 and double asterisks (**) indicate *P*<.01.

Figure 2 illustrates the combined effects of solid fuel use and circadian rhythm syndrome on physical function and muscle strength. Specifically, participants who used solid fuels and had circadian rhythm syndrome concurrently (solid fuels + circadian rhythm syndrome group) had the most pronounced reduction in physical function (*β*=–0.698, 95% CI –0.813 to –0.584) and muscle strength (*β*=–0.332, 95% CI –0.387 to –0.277) compared with those who did not use solid fuels or had only one of the risk factors.

### Stratified Analyses

**Figure 3:**
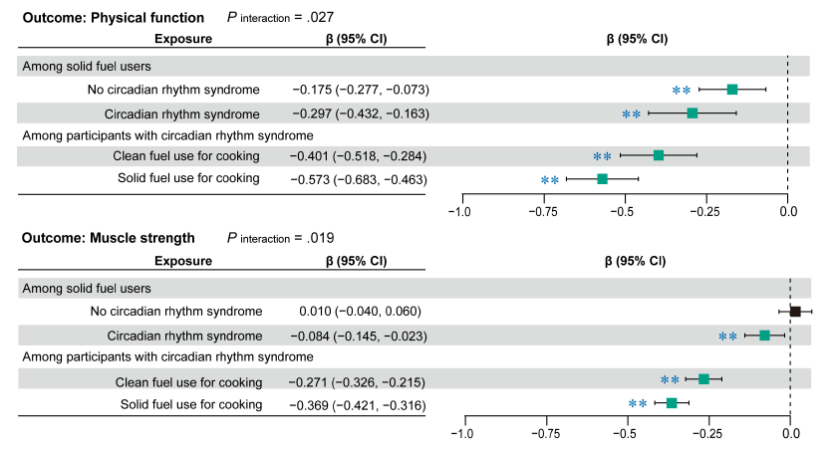
Stratified analyses were conducted separately for circadian rhythm syndrome and household fuel types. Estimates were derived from multivariable linear regression models stratified by fuel type and circadian rhythm syndrome. Adjusted for age, gender, BMI, urbanization, education level, marital status, smoking status, drinking status, housing type, indoor temperature, and chronic diseases. A single asterisk (*) indicates *P*<.05 and double asterisks (**) indicate *P*<.01.

As we detected a multiplicative interaction between solid fuel use and circadian rhythm syndrome on physical function (*P*_interaction_=.03) and muscle strength (*P*_interaction_=.02), we conducted further stratified analyses (Figure 3). Our findings indicated that individuals with circadian rhythm syndrome were more vulnerable to the effects of solid fuel use on physical function (*β*=–0.297, 95% CI –0.432 to –0.163) and muscle strength (*β*=–0.084, 95% CI –0.145 to –0.023). Similarly, the negative correlation between circadian rhythm syndrome and physical function (*β*=–0.573, 95% CI –0.683 to –0.463) and muscle strength (*β*=–0.369, 95% CI –0.421 to –0.316) was more pronounced in participants who used solid fuels.

### Mediation Analysis

**Figure 4:**
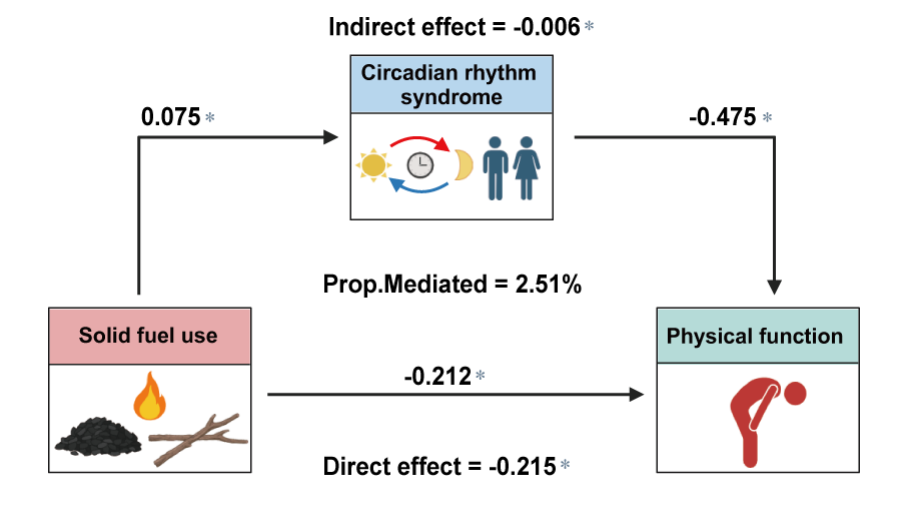
Mediation analysis of the association among solid fuel use, circadian rhythm syndrome, and physical function. Mediation analysis was conducted using the R package mediation to examine whether circadian rhythm syndrome mediated the association between solid fuel use and physical function scores. Regression models were fitted for both the mediator and the outcome, adjusting for age, gender, BMI, urbanization, education level, marital status, smoking status, drinking status, housing type, indoor temperature, and chronic diseases. Direct, indirect, and total effects were estimated using 1000 bootstrap simulations to derive 95% CIs. A single asterisk (*) indicates *P*<.05.

The results of the mediation analysis are depicted in Figure 4. Circadian rhythm syndrome served as a significant mediator of the positive association between solid fuel use and physical function, with a mediation effect of 2.51%.

### Sensitivity Analyses

Multiple sensitivity analyses were performed to verify the reliability and validity of the findings. When examining the association between solid fuel use and the number of circadian rhythm syndrome components, we found that participants who used solid fuels for both cooking and heating exhibited a 5.5% increase in the number of syndrome components compared with those using clean fuels (Multimedia Appendix 10). We also examined the effects of different types of solid fuels on physical function and circadian rhythm syndrome and found that coal use was more strongly correlated with both physical function (*β*=*–*0.276, 95% CI *–*0.411 to *–*0.141) and circadian rhythm syndrome (OR 1.221, 95% CI 1.02 to 1.463), whereas crop residue/wood burning was only significantly associated with physical function (*β*=–0.198, 95% CI –0.286 to –0.11) (Multimedia Appendices 11 and 12). The results of the subgroup analyses, shown in Multimedia Appendix 13, indicate that older adults are more susceptible to the impact of circadian rhythm syndrome on physical function scores, highlighting the importance of maintaining regular work and rest habits in this population. We excluded participants who reported “other” as their fuel type and reexamined the main associations among solid fuel use, circadian rhythm syndrome, and physical function in both cross-sectional and longitudinal analyses. The results were consistent with the primary findings (Multimedia Appendices 14-16), supporting the robustness of the observed associations.

## Discussion

### Principal Findings and Comparison With Prior Works

This study found that solid fuel use was positively associated with circadian rhythm syndrome, and that both solid fuel use and circadian rhythm syndrome were independently and jointly associated with poorer physical function and lower muscle strength. A significant multiplicative interaction was observed, indicating that the associations between solid fuel use and these functional outcomes were stronger among participants with circadian rhythm syndrome. In addition, circadian rhythm syndrome partially mediated the association between solid fuel use and physical function, suggesting that circadian rhythm disruption may represent one potential pathway underlying the observed associations.

To the best of our knowledge, this is the first study to investigate the relationship between household air pollution from solid fuel use and circadian rhythm syndrome, making it difficult to compare our results with those of previous studies. Although previous studies have not reported a direct link between solid fuel use and circadian rhythm syndrome, associations between solid fuel use and various components of the circadian rhythm syndrome have been demonstrated. Results from a cross-sectional study involving women suggested that household air pollution from burning biomass fuel is associated with an increased risk of metabolic syndrome [29]. Studies from different regions have consistently confirmed the association between household air pollution and components of metabolic syndrome, such as blood pressure, blood glucose, and lipid profile, highlighting the potential risk of metabolic disorders that can be reduced by mitigating household air pollution [30-32]. The use of solid fuels also affects the mental health of older adults, with the results of a cohort study showing that using solid fuels for heating and cooking increases the risk of depression by 45% [33]. Similarly, the sleep-related health of middle-aged and older adults is affected by the use of solid fuels, especially in rural areas, where the vast majority of households rely on the combustion of biomass fuels for nighttime heating in winter. Studies from different regions have shown a significant positive association among solid fuel use, poor sleep quality, and reduced sleep duration [34,35]. Metabolic homeostasis, social stress, and sleep significantly affect the stability of the circadian clock, and exploring the effects of solid fuel use on one of them alone is one-sided. Therefore, this study examined the comprehensive impact of household air pollution on circadian rhythm disorders and revealed the importance of household fuel cleanliness on circadian rhythm homeostasis in middle-aged and older adults (OR 1.315, 95% CI 1.155 to 1.499 at baseline; OR 1.078, 95% CI 1.031 to 1.125 at follow-up). We also further analyzed the impact of household air pollution on circadian rhythm syndrome components; participants who used solid fuels for heating and cooking showed greater accumulation of circadian rhythm syndrome components than participants who used clean fuels. Cooking and heating are 2 common and often coexisting household activities; therefore, examining the independent and combined use of solid fuels in these settings is of particular relevance. We found that, compared with the use of clean fuels for both purposes, using solid fuels for either cooking or heating, as well as for both, was associated with a higher likelihood of circadian rhythm syndrome (either cooking or heating: OR 1.172, 95% CI 1.024 to 1.341; both: OR 1.315, 95% CI: 1.155 to 1.499) and lower physical function scores (either cooking or heating: *β*=–0.175, 95% CI –0.293 to –0.057; both: *β*=–0.386, 95% CI –0.5 to –0.272) in a graded manner. These findings suggest that different patterns of household fuel exposure may exert additive adverse effects on circadian regulation and physical function.

Disturbances in the circadian rhythm pose a major threat to the health of the musculoskeletal system in middle-aged and older adults. Real-life problems faced by older adults, such as a reduced metabolic rate, sleep disturbances, and depression caused by loneliness, affect the stability of circadian rhythms and impair muscle, joint, and bone health [9,36]. Pathophysiological changes in the musculoskeletal system significantly affect mobility and muscle strength and accelerate aging, which in turn leads to the disruption of circadian rhythms [3,37]. Although the vicious cycle of circadian rhythm syndrome and reduced physical function and muscle strength is detrimental to the health of middle-aged and older adults, no high-quality epidemiological studies have confirmed this association. Using a large nationwide cohort, we observed a significant inverse association among circadian rhythm syndrome, physical function (*β*=–0.386, 95% CI –0.5 to –0.272 at baseline; *β*=–0.475, 95% CI –0.555 to –0.394 at follow-up), and muscle strength (*β*=–0.061, 95% CI –0.117 to –0.005 at baseline; *β*=–0.304, 95% CI –0.342 to –0.265 at follow-up). We also evaluated the impact of circadian rhythm syndrome on physical performance (assessed by gait speed and chair stand tests) and showed that disruption of the circadian rhythm impairs mobility in middle-aged and older adults.

The decline in muscle strength and mobility due to aging causes most middle-aged and older adults to stay indoors for long periods. Therefore, it is important to explore the comprehensive impacts of indoor environmental factors and lifestyle. We found that solid fuel use and circadian rhythm syndrome had a synergistic effect on the impairment of physical function (*β*=–0.698, 95% CI –0.813 to –0.584) and muscle strength (*β*=–0.332, 95% CI –0.387 to –0.277). There was a multiplicative interaction between solid fuel use and circadian rhythm syndrome (*P*_interaction_<.05), with circadian rhythm syndrome exacerbating the effects of solid fuel use on physical function (*β*=–0.297, 95% CI –0.432 to –0.163) and muscle strength (*β*=–0.084, 95% CI –0.145 to –0.023). Since middle-aged and older adults, especially in rural areas, mostly use solid fuels for nighttime heating, sleep disorders caused by circadian rhythm disorders are more likely to be exposed to household air pollution caused by solid fuels, which in turn leads to a more significant negative correlation with physical function and muscle strength. Furthermore, we elucidated the mediating role of circadian rhythm syndrome, whereby solid fuel use impaired physical function in part by increasing the risk of circadian rhythm syndrome (mediation effect: 2.51%).

Mechanistically, disruption of central and peripheral circadian regulatory networks may underlie the associations observed among solid fuel use and impaired physical function and muscle strength. The combustion of solid fuels generates particulate matter and toxic gases, which have been shown to enter systemic circulation and may affect the central nervous system, potentially disrupting the coordination between the central pacemaker in the suprachiasmatic nucleus and peripheral clocks through neurohumoral pathways [38-40]. In addition, household air pollution has been associated with increased systemic inflammation and oxidative stress. Air pollutants can stimulate the production of inflammatory cytokines and reactive oxygen species while impairing antioxidant defense systems [41,42]. These processes may contribute to circadian dysregulation, as both inflammation and oxidative stress have been implicated in altering clock gene expression and circadian homeostasis. Furthermore, chronic inflammation and oxidative stress are known to adversely affect joint integrity and muscle function, thereby contributing to declines in physical function [12]. Metabolic pathways may also play a role in linking circadian disruption to functional impairment. Experimental studies have shown that disturbances in skeletal muscle circadian rhythms can lead to metabolic dysregulation, including increased reliance on anaerobic glycolysis, reduced mitochondrial function, and increased apoptosis [9,43]. Such changes may accelerate muscle degradation and contribute to sarcopenia. Taken together, although the precise mechanisms remain to be fully elucidated, existing evidence suggests that household air pollution from solid fuel use may influence circadian regulation through interconnected pathways involving neurohumoral disruption, systemic inflammation, oxidative stress, and metabolic imbalance, ultimately contributing to impaired physical function and reduced muscle strength. Consequently, although the precise mechanisms remain unclear, the relationship between household air pollution due to solid fuel use, circadian rhythm syndrome, physical function, and muscle strength warrants further investigation.

This study has several strengths: (1) novel findings, we identified an association between solid fuel use and circadian rhythm syndrome, with both solid fuel use and circadian rhythm syndrome separately and synergistically associated with declines in physical function and muscular strength; (2) representative and reliable results, we used a large national cohort of 126 cities across 28 provinces to conduct meticulous sensitivity analyses; (3) rigorous analytic methods, to minimize the recall and social desirability biases inherent in self-report measures, we used repeated-measures tests focusing on physical function, muscular strength, and physical performance in middle-aged and older adults; (4) guiding insights, mediation analyses revealed that solid fuel use affected physical function and muscular strength by increasing the risk of circadian rhythm syndrome, emphasizing the importance of household fuel cleaning and regular work and rest habits for the health of middle-aged and older adults.

### Limitations

However, some limitations of this study should be noted. The CHARLS is a representative cohort study of middle-aged and older individuals. We only evaluated the impact of circadian rhythm syndrome on the health of middle-aged and older adults. The differences between the different age groups warrant further exploration. In addition, information on household solid fuel use was self-reported by participants, which may be subject to recall bias or reporting bias. This study relied solely on information on household fuel use for heating and cooking to assess indoor air pollution and may have overlooked other factors, such as ventilation efficiency and weather conditions. The degree of dependence on solid fuels varies seasonally. For example, in winter, the dependence on solid fuels for heating is higher. It is necessary to pay attention to the differences in the use of solid fuels in different seasons with respect to circadian rhythm syndrome, physical function, and muscle strength. Moreover, given the observational nature of this study, the identified associations among solid fuel use, circadian rhythm syndrome, and physical function or muscle strength should be interpreted with caution. Although we adjusted for a wide range of potential confounders, residual confounding cannot be completely ruled out, and causal relationships cannot be definitively established.

In conclusion, this nationwide prospective cohort study revealed a positive association between solid fuel use and circadian rhythm syndrome and found negative effects of solid fuel use and circadian rhythm syndrome, separately and jointly, on physical function and muscle strength. Disturbances in the circadian rhythm exacerbate the decline in physical function and muscle strength caused by solid fuel use. Circadian rhythm syndrome is an important mediator in the relationship between solid fuel use and physical function. This study highlights the combined impact of indoor environmental factors and lifestyle on middle-aged and older adults and recommends the use of clean fuels and the development of regular work and rest habits to improve physical health.

## Appendix

Multimedia Appendix 1 Selection process of participants.

Multimedia Appendix 2 Spatial distribution of participants.

Multimedia Appendix 3 Assessment criteria and definition of circadian rhythm syndrome.

Multimedia Appendix 4 Supplementary methods for the study.

Multimedia Appendix 5 Comparison of basic information between the original dataset and the dataset used in the study. Continuous and categorical variables were compared using analyses of independent sample Student *t* test and chi-square tests, respectively.

Multimedia Appendix 6 Summary characteristics of 5018 participants in longitudinal analysis.

Multimedia Appendix 7 Spatial distribution of solid fuel use in households, scores of physical function and grip strength, and circadian rhythm syndrome participants Notes: Spatial distributions were estimated using ordinary kriging interpolation to construct a continuous prediction surface from observed provincial data. Spatial patterns were visualized using ordinary kriging interpolation based on observed CHARLS data. Tibet, Ningxia, Hainan, and Taiwan had no original participants in the analytic dataset; therefore, the values shown for these regions are interpolated estimates rather than observed data, whereas all other provinces reflect distributions based on observed data. The interpolated estimates were derived from spatial autocorrelation patterns of neighboring provinces. Color gradients represent predicted levels of the outcome variable.

Multimedia Appendix 8 Effects of solid fuel use on four test scores of physical functions after adjusting for all covariates. Notes: Adjusted for age, gender, BMI, urbanization, education level, marital status, smoking status, drinking status, housing type and indoor temperature, and chronic diseases.

Multimedia Appendix 9 Effects of solid fuel use and circadian rhythm syndrome on four test scores of physical functions after adjusting for all covariates. Notes: Adjusted for age, gender, BMI, urbanization, education level, marital status, smoking status, drinking status, housing type and indoor temperature, and chronic diseases.

Multimedia Appendix 10 Association between household solid fuel use and number of circadian rhythm syndrome components: negative binomial regression models. Notes: Adjusted for age, gender, BMI, urbanization, education level, marital status, smoking status, drinking status, housing type and indoor temperature, and chronic diseases. IRR: incidence rate ratio.

Multimedia Appendix 11 Effects of different solid fuel types on physical function and circadian rhythm syndrome. Notes: Adjusted for age, gender, BMI, urbanization, education level, marital status, smoking status, drinking status, housing type and indoor temperature, and chronic diseases.

Multimedia Appendix 12 Effects of solid fuel use on physical function in participants for whom data were available in 2011, 2013, and 2015. Notes: Adjusted for age, gender, BMI, urbanization, education level, marital status, smoking status, drinking status, housing type and indoor temperature, and chronic diseases.

Multimedia Appendix 13 Subgroup analysis of effects of solid fuel use and circadian rhythm syndrome on physical function. Notes: All models were adjusted for age, gender, BMI, urbanization, education level, marital status, smoking status, drinking status, housing type and indoor temperature, and chronic diseases, except for the stratifying variables.

Multimedia Appendix 14 Sensitivity analysis excluding participants reporting “Other” fuel type: cross-sectional associations among solid fuel use, circadian rhythm syndrome, and physical function. Notes: Estimates were derived from multivariable regression models (binary logistic regression for circadian rhythm syndrome and linear models for cross-sectional analyses). Results shown are from model 4, adjusted for age, gender, BMI, urbanization, education level, marital status, smoking status, drinking status, housing type and indoor temperature, and chronic diseases.

Multimedia Appendix 15 Sensitivity analysis excluding participants reporting “Other” fuel type: Longitudinal associations among solid fuel use, circadian rhythm syndrome, and physical function. Notes: Estimates were derived from mixed-effects linear models for longitudinal analyses. Results shown are from model 4, adjusted for age, gender, BMI, urbanization, education level, marital status, smoking status, drinking status, housing type and indoor temperature, and chronic diseases.

Multimedia Appendix 16 Sensitivity analysis excluding participants reporting “Other” fuel type: combined associations among solid fuel use, circadian rhythm syndrome, and physical function. Notes: Estimates were derived from multivariable linear regression models including a 2 × 2 combined exposure variable (fuel type × circadian rhythm syndrome) and a multiplicative interaction term to assess interaction effects. Adjusted for age, gender, BMI, urbanization, education level, marital status, smoking status, drinking status, housing type and indoor temperature, and chronic diseases. Circs: circadian rhythm syndrome.

## Abbreviations

CHARLS: China Health and Retirement Longitudinal Study
OR: odds ratio

## Data Availability

The datasets generated or analyzed during this study are available from the corresponding author on reasonable request.
