# Supplementary figures and images for: Associations of Solid Fuel Use and Circadian Rhythm Syndrome With Physical Function and Muscle Strength in Middle-Aged and Older Adults: Nationwide Cohort Study in China
Source: JMIR Aging. 2026 Jun 29;9:e78352. doi: 10.2196/78352 (PMC13365896; doi:10.2196/78352)

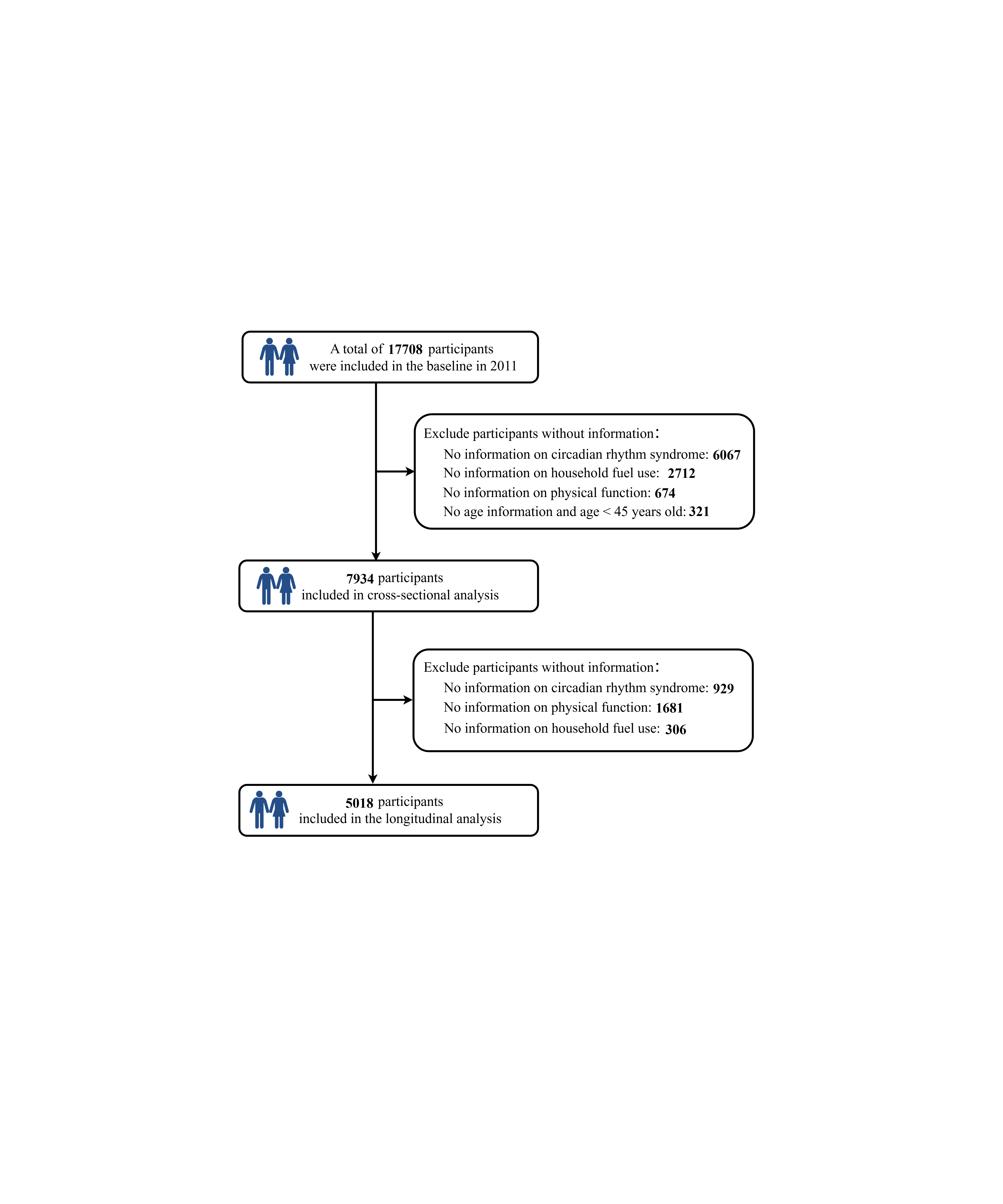

Supplement: Multimedia Appendix 1 [file aging_v9i1e78352_app1.png]

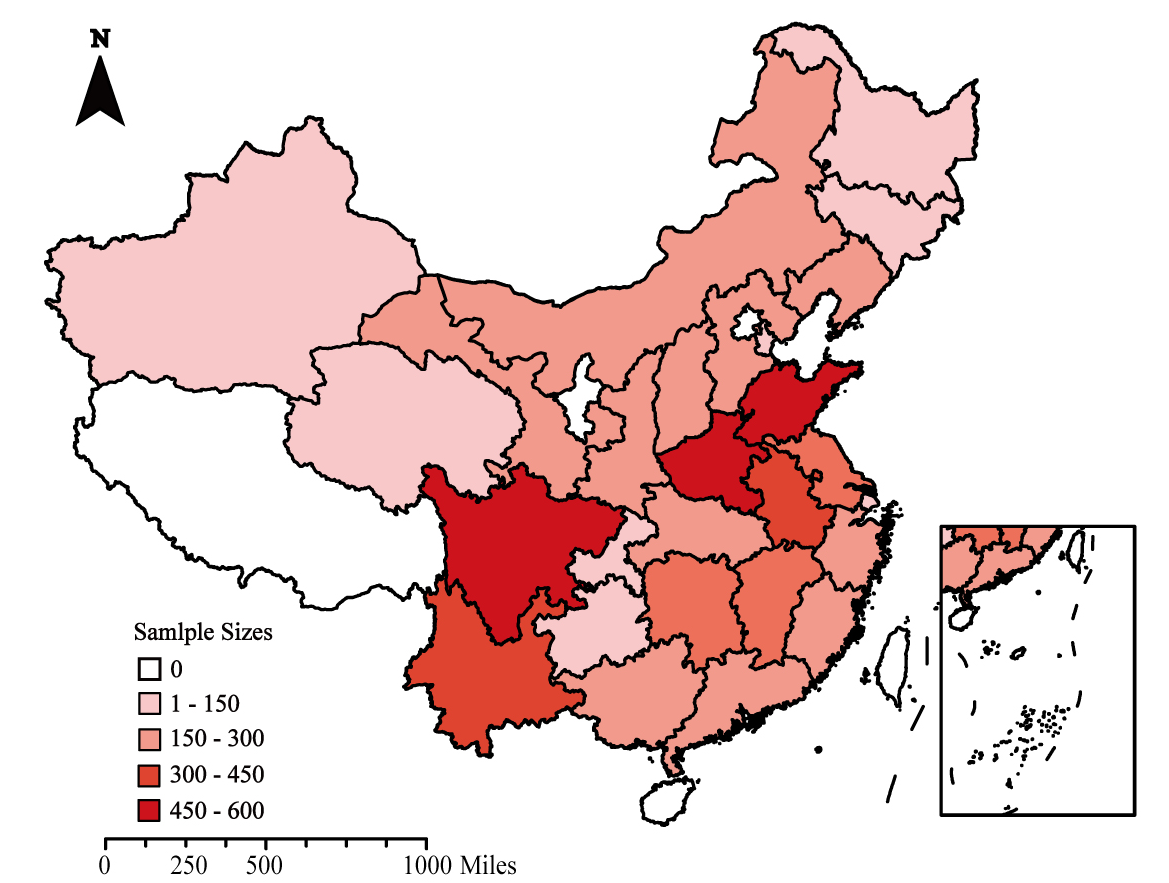

Supplement: Multimedia Appendix 2 [file aging_v9i1e78352_app2.png]

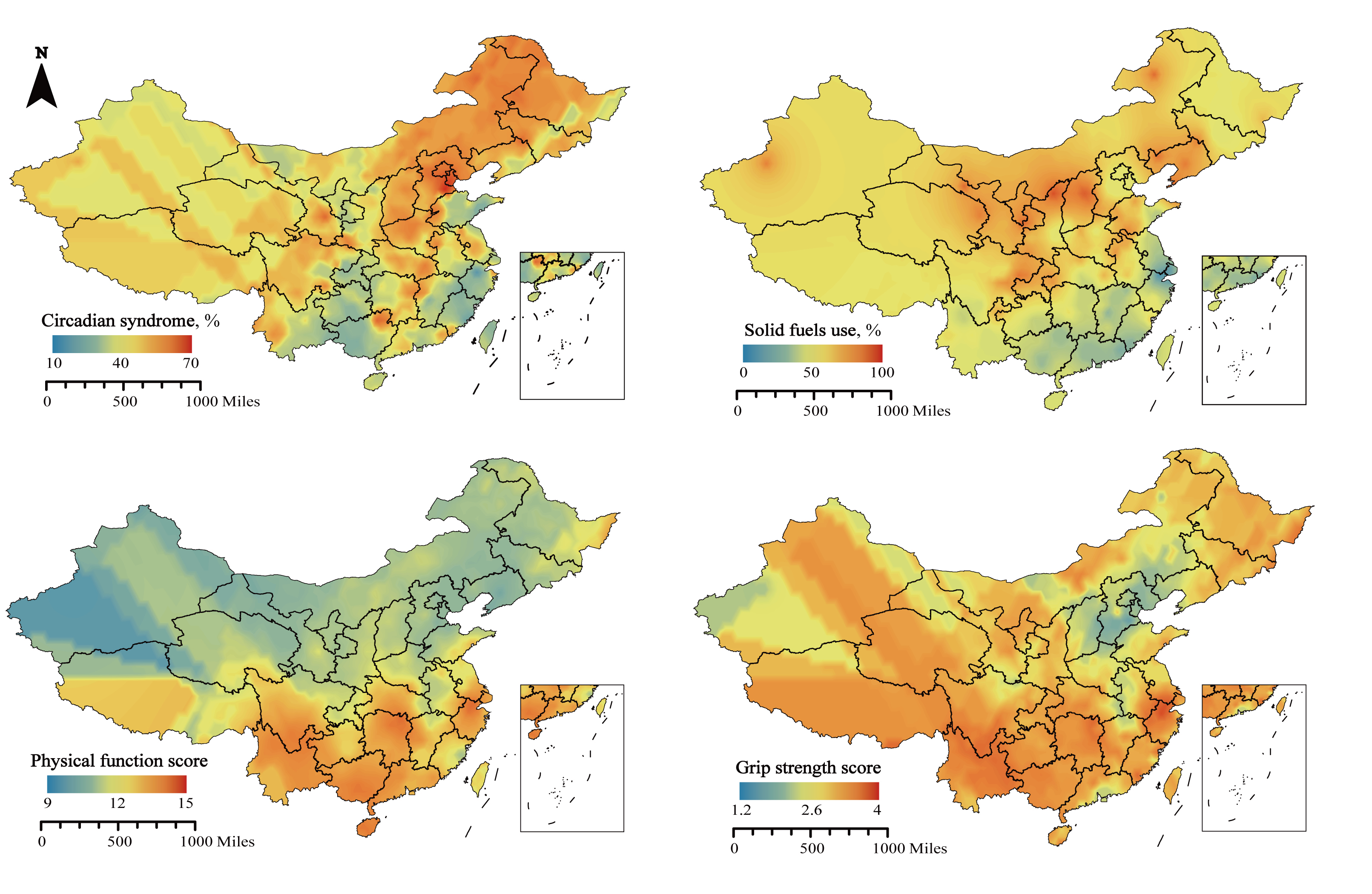

Supplement: Multimedia Appendix 7 [file aging_v9i1e78352_app7.png]

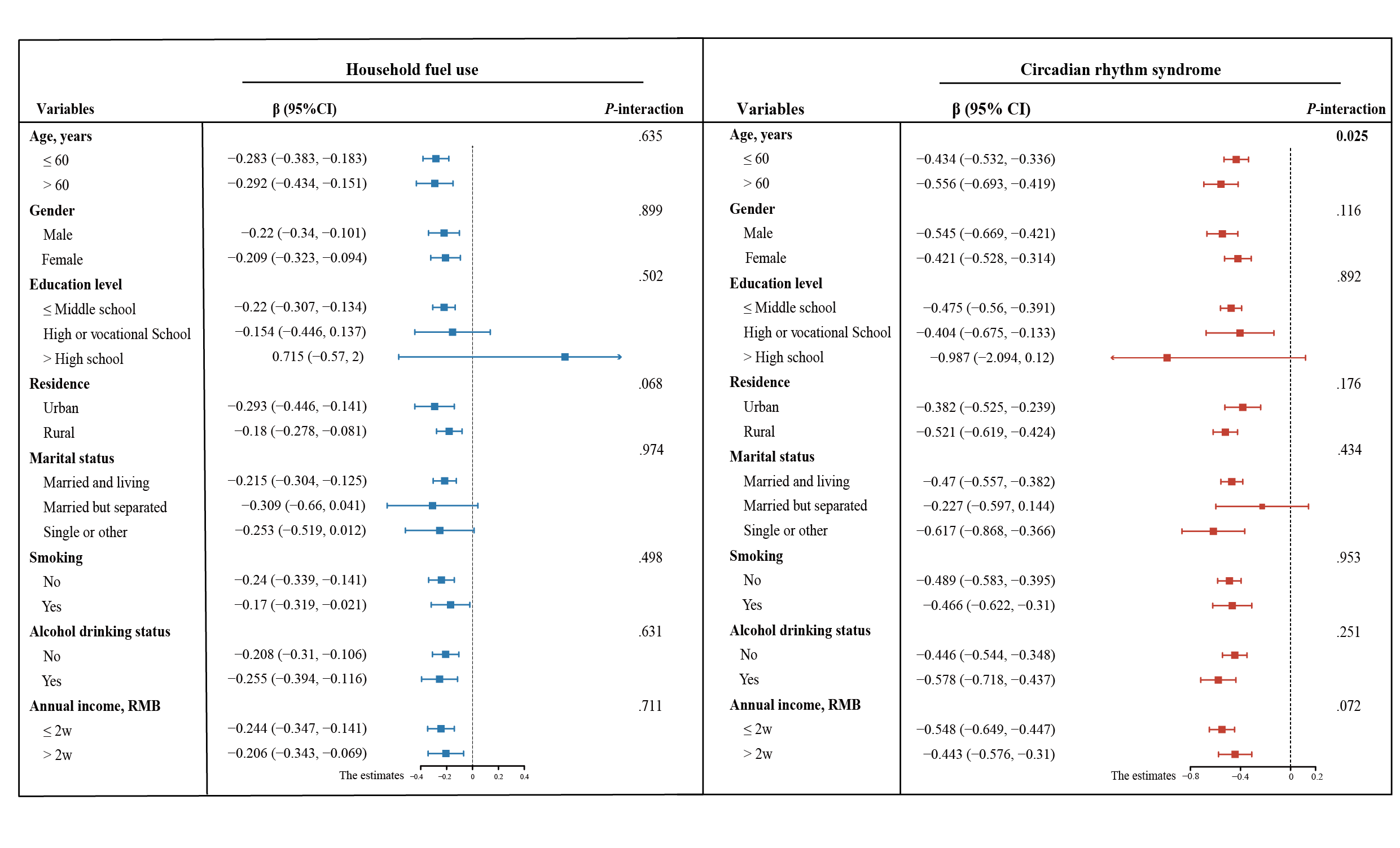

Supplement: Multimedia Appendix 13 [file aging_v9i1e78352_app13.png]
