# Supplementary material for: Associations of Solid Fuel Use and Circadian Rhythm Syndrome With Physical Function and Muscle Strength in Middle-Aged and Older Adults: Nationwide Cohort Study in China
Source: JMIR Aging. 2026 Jun 29;9:e78352. doi: 10.2196/78352 (PMC13365896; doi:10.2196/78352)
Supplement: Multimedia Appendix 3 [file aging_v9i1e78352_app3.pdf]

| Component | Diagnostic Criteria |
|---|---|
| **Elevated blood pressure**[a] | 1. Systolic blood pressure ≥ 130 mmHg and/or diastolic blood pressure ≥ 85 mmHg 2. Diagnosed with hypertension by a doctor 3. Taking medication for hypertension |
| **Hyperglycemia**[a] | 1. Fasting plasma glucose ≥ 100 mg/dL 2. Diagnosed with diabetes by a doctor 3. Taking medication for hyperglycemia |
| **Elevated triglycerides**[a] | 1. Venous blood ≥ 150 mg/dL 2. Taking medication for triglyceride abnormality |
| **Elevated waist circumference** | ≥ 85 cm in men or ≥ 80 cm in women |
| **Reduced HDL-C**[a] | 1. Venous blood < 40 mg/dL in men or < 50 mg/dL in women 2. Taking medication for HDL-C abnormality |
| **Short sleep duration** | Night sleep duration of < 6 hours[c] |
| **Depression** | CES-D score ≥ 10[d] |

**Notes:** HDL-C, HDL-cholesterol. CES-D, Center for Epidemiological Studies Depression Scale.

[a]Participants who met any of these criteria were diagnosed.

[b]Blood pressure was recorded on the right upper arm by trained staff using an Omron™ HEM-7112 blood pressure monitors at 45-second intervals after 5 minutes of rest in a seated position. The average of the three measurements was used as the blood pressure value.

[c]According to the National Sleep Foundation's definition, short sleep duration was defined as a self-reported night sleep duration of <6 hours [1].

[d]Depression was assessed using the 10-question Center for Epidemiological Studies Depression Scale (CES-D), and participants with a CES-D score ≥ 10 were defined as depressed [2].

**References:**


1. M. Hirshkowitz, K. Whiton, S. M. Albert, C. Alessi, O. Bruni, L. DonCarlos, N. Hazen, J. Herman, E. S. Katz, L. Kheirandish-Gozal, D. N. Neubauer, A. E. O'Donnell, M. Ohayon, J. Peever, R. Rawding, R. C. Sachdeva, B. Setters, M. V. Vitiello, J. C. Ware, P. J. Adams Hillard. National Sleep Foundation's sleep time duration recommendations: methodology and results summary. Sleep Health 2015; 1(1) 40-43.

2. E. M. Andresen, J. A. Malmgren, W. B. Carter, D. L. Patrick. Screening for depression in well older adults: evaluation of a short form of the CES-D (Center for Epidemiologic Studies Depression Scale). Am J Prev Med 1994; 10(2) 77-84.
