# Supplementary material for: Associations of Solid Fuel Use and Circadian Rhythm Syndrome With Physical Function and Muscle Strength in Middle-Aged and Older Adults: Nationwide Cohort Study in China
Source: JMIR Aging. 2026 Jun 29;9:e78352. doi: 10.2196/78352 (PMC13365896; doi:10.2196/78352)
Supplement: Multimedia Appendix 4 [file aging_v9i1e78352_app4.pdf]

Covariates

Age, gender (male, female), education level (≤ middle school, high or vocational school, > high school), marital status (married and living, married but separated, single or other), and urbanization level (urban, rural). In addition, we adjusted for personal habits as well as economic and health status to analyze the associations between solid fuel use, circadian rhythm syndrome, physical function, and muscle strength: smoking status (never, ever), drinking status (never, current), annual income (≤ 20000, > 20000), indoor temperature (hot, bearable, cold), housing type (one-story, multi-story), body mass index (BMI), and a comprehensive set of chronic diseases (yes, no), including cancer, chronic lung disease, heart problem, stroke, psychiatric problems, arthritis, liver disease, kidney disease, digestive disease, asthma, and memory disorder.

Statistical analysis

Cross-sectional And Longitudinal Analysis of Separate Effects

We examined the relationships among solid fuel use, circadian rhythm syndrome, and physical function scores in both cross-sectional and longitudinal studies. First, we employed binary logistic regression models to assess the relationship between solid fuel use and circadian rhythm syndrome by calculating odds ratios (ORs) and corresponding 95% confidence intervals (CIs). Considering that circadian rhythm syndrome comprises seven components, we used negative binomial regression models to examine the association between solid fuel use and the number of circadian rhythm syndrome components. Subsequently, in order to explore the associations of solid fuel and circadian rhythm syndrome with physical function scores, we utilized linear models in cross-sectional analysis

and mixed-effects linear models in longitudinal analysis, estimating their regression coefficients (β) and corresponding 95% CIs. Given the potential influence of the covariates on the effects, we incrementally introduced them into the four models. In Model 1, solid fuel use and circadian rhythm syndrome were treated as fixed-effect terms, individual ID as a random effect, and survey time was included as a categorical variable (2011 and 2015) to control for differences across survey times. Model 2 was adjusted for the Model 1 covariates, age, sex, and BMI. Model 3 was adjusted for the Model 2 covariates, urbanization level, educational level, marital status, smoking status, drinking status, housing type, and indoor temperature. Model 4 was adjusted for various chronic diseases.

Mediation Analysis

We conducted a mediation analysis using the R package "mediation" to assess whether circadian rhythm syndrome mediates the association between solid fuel use and physical function scores. The total effect of solid fuel use was decomposed into direct and indirect effects via circadian rhythm syndrome. Regression models were first fitted for the mediator and the outcome, adjusting for relevant covariates. Mediation effects, including the average causal mediation effect, average direct effect, and total effect, were estimated with 1,000 bootstrap simulations to derive 95% confidence intervals.

Sensitivity Analyses

To evaluate the robustness of our primary findings, we conducted several sensitivity analyses. First, we evaluated the impact on the primary association after removing participants who reported "other" solid fuel types. Moreover, we conducted subgroup analyses to assess potential heterogeneity in the associations of solid fuel use and circadian

rhythm syndrome across different population subgroups. Additionally, we examined the associations between different numbers of circadian rhythm syndrome components and physical function scores. Furthermore, we examined coal use and crop residue/wood burning separately while also considering the multiple fuels utilized in households within the China Health and Retirement Longitudinal Study. We excluded circadian rhythm syndrome data for the year 2013 from our analyses because it was unavailable. However, data on physical function from 2013 were accessible. Consequently, participants with physical function data for 2011, 2013, and 2015 were included in the study, allowing for a reassessment of the effect of solid fuel on physical function scores.
