# Supplementary material for: Associations of Solid Fuel Use and Circadian Rhythm Syndrome With Physical Function and Muscle Strength in Middle-Aged and Older Adults: Nationwide Cohort Study in China
Source: JMIR Aging. 2026 Jun 29;9:e78352. doi: 10.2196/78352 (PMC13365896; doi:10.2196/78352)
Supplement: Multimedia Appendix 5 [file aging_v9i1e78352_app5.pdf]

Variable	Original	Imputed	*P* value [a]	Variable	Original	Imputed	*P* value [a]
**Demographic characteristics, n (%)**				Unknown	142 (1.1)	0	
Smoking status			.996				
Never	9060 (70)	9061 (70)		**Health status, n (%)**	1544 (11.9)	1556 (12)	
Ever	3890 (30)	3891 (30)		Chronic lung disease	1544 (11.9)	1556 (12)	.973
Unknown	2 (< 0.1)	0		Unknown	85 (0.7)	0	
Alcohol drinking status			.996	Heart problem	1784 (13.8)	1784 (13.8)	.749
Never	8622 (66.6)	8623 (66.6)		Unknown	128 (1)	0	
Ever	4329 (33.4)	4329 (33.4)		Stroke	338 (2.6)	340 (2.6)	.979
Unknown	1 (< 0.1)	0		Unknown	51 (0.4)	0	
				Psychiatric problems	191 (1.5)	199 (1.5)	.73
**Household characteristics, n (%)**				Unknown	81 (0.6)	0	
Housing type			.862	Arthritis	5123 (39.6)	5151 (39.8)	.969
One-story	8194 (63.3)	8231 (63.6)		Unknown	78 (0.6)	0	
Multi-story	4721 (36.4)	4721 (36.4)		Liver disease	590 (4.6)	590 (4.6)	.844
Unknown	37 (0.3)	0		Unknown	144 (1.1)	0	
Indoor temperature			.991	Kidney disease	997 (7.7)	997 (7.7)	.824
Bearable	10965 (84.7)	11094 (85.7)		Unknown	123 (0.9)	0	
Hot	1418 (10.9)	1428 (11)		Digestive disease	3498 (27)	3531 (27.3)	.884
Cold	427 (3.3)	430 (3.3)		Unknown	83 (0.6)	0	
Unknown	142 (1.1)	0		Asthma	719 (5.6)	719 (5.6)	.921
				Unknown	66 (0.5)	0	

							(continued)
**Health status, n (%)**				Memory disorder	209 (1.6)	209 (1.6)	.96
Cancer	12707 (98.1)	142 (1.1)	.946	Unknown	63 (0.5)	0	
