# Supplementary material for: Associations of Solid Fuel Use and Circadian Rhythm Syndrome With Physical Function and Muscle Strength in Middle-Aged and Older Adults: Nationwide Cohort Study in China
Source: JMIR Aging. 2026 Jun 29;9:e78352. doi: 10.2196/78352 (PMC13365896; doi:10.2196/78352)
Supplement: Multimedia Appendix 6 [file aging_v9i1e78352_app6.pdf]

Variable	Wave 1	Wave 3	Variable	Wave 1	Wave 3
**Observations, n**	5018	5018	> 20000	1938 (38.6)	1438 (28.7)
**Demographic characteristics, n (%)**			**Health status, n (%)**		
Age, mean (SD), years	58.1 (8.3)	62.1 (8.3)	BMI, mean (SD), kg/m^2	23.9 (13.1)	24.5 (18.9)
Gender			Cancer	36 (0.7)	76 (1.5)
Male	2370 (47.2)	2370 (47.2)	Chronic lung disease	494 (9.8)	752 (15)
Female	2648 (52.8)	2648 (52.8)	Heart problem	588 (11.7)	905 (18)
Education level			Stroke	93 (1.9)	166 (3.3)
≤ Middle school	4623 (92.1)	4623 (92.1)	Psychiatric problems	53 (1.1)	107 (2.1)
High or vocational School	361 (7.2)	361 (7.2)	Arthritis	1724 (34.4)	2364 (47.1)
> High school	34 (0.7)	34 (0.7)	Dyslipidemia	500 (10)	989 (19.7)
Residence			Liver disease	155 (3.1)	325 (6.5)
Urban	1563 (31.1)	1563 (31.1)	Kidney disease	300 (6)	527 (10.5)
Rural	3455 (68.9)	3455 (68.9)	Digestive disease	1165 (23.2)	1675 (33.4)
Marital status			Asthma	244 (4.9)	344 (6.9)
Married and living	4342 (86.5)	4165 (83)	Memory disorder	51 (1)	110 (2.2)
Married but separated	211 (4.2)	205 (4.1)	**Circadian rhythm syndrome**	1928 (38.4)	1840 (36.7)
Single or other	465 (9.3)	648 (12.9)	**Circadian rhythm syndrome component, n (%)**		
Smoking status			Reduced HDL-C	1993 (39.7)	1789 (35.7)
Never	3455 (68.9)	3634 (72.4)	Elevated waist circumference	2909 (58)	3127 (62.3)
Ever	1563 (31.1)	1384 (27.6)	Hyperglycemia	2867 (57.1)	1845 (36.8)

					(continued)
Alcohol drinking status			Raised blood pressure	2910 (58)	3014 (60.1)
Never	3351 (66.8)	3334 (66.4)	Elevated triglyceride	1442 (28.7)	1796 (35.8)
Ever	1667 (33.2)	1684 (33.6)	Short sleep duration	1460 (29.1)	1581 (31.5)
**Household characteristics, n (%)**			Depressive symptoms	1863 (37.1)	1742 (34.7)
Housing type			**Physical function, mean (SD)**		
One-story	3448 (68.7)	2864 (57.1)	Physical function score	12.3 (2.3)	11.9 (2.5)
Multi-story	1570 (31.3)	2154 (42.9)	Muscle strength score	2.5 (1.1)	2.5 (1.1)
Indoor temperature			Gait speed score	3.4 (1)	3.1 (1.1)
Bearable	4238 (84.5)	4435 (88.4)	Chair stand test score	2.5 (1.1)	2.5 (1.1)
Hot	608 (12.1)	424 (8.4)	Balance score	3.9 (0.4)	3.9 (0.5)
Cold	172 (3.4)	159 (3.2)	**Household fuel use, n (%)**		
Annual income, RMB			Clean	1915 (38.2)	2581 (51.4)
≤ 20000	3080 (61.4)	3580 (71.3)	Solid	3103 (61.8)	2437 (48.6)
