# Supplementary material for: Associations of Solid Fuel Use and Circadian Rhythm Syndrome With Physical Function and Muscle Strength in Middle-Aged and Older Adults: Nationwide Cohort Study in China
Source: JMIR Aging. 2026 Jun 29;9:e78352. doi: 10.2196/78352 (PMC13365896; doi:10.2196/78352)
Supplement: Multimedia Appendix 8 [file aging_v9i1e78352_app8.pdf]

Type	Muscle strength		Gait speed	Chair stand test		Balance	
	β (95%CI)		β (95%CI)	β (95%CI)		β (95%CI)	
**Household fuel use**							
Individual effects							
Cooking fuel use							
Clean fuels	0 (Reference)		0 (Reference)	0 (Reference)		0 (Reference)	
Solid fuels	-0.009 (-0.054, 0.036)		-0.031 (-0.07, 0.008)	-0.126 (-0.176, -0.075)	*	-0.024 (-0.046, -0.001)	*
Heating fuel use							
Clean fuels	0 (Reference)		0 (Reference)	0 (Reference)		0 (Reference)	
Solid fuels	-0.07 (-0.113, -0.026)	**	-0.017 (-0.055, 0.02)	-0.233 (-0.281, -0.184)	**	0.003 (-0.019, 0.024)	
Combined effects							
Clean fuel use for both cooking and heating	0 (Reference)		0 (Reference)	0 (Reference)		0 (Reference)	
Solid fuel use for either cooking or heating	-0.026 (-0.084, 0.032)		-0.033 (-0.083, 0.017)	-0.123 (-0.188, -0.058)	**	0.006 (-0.022, 0.035)	
Solid fuel use for both cooking and heating	-0.061 (-0.117, -0.005)	*	-0.039 (-0.088, 0.009)	-0.273 (-0.335, -0.211)	**	-0.013 (-0.04, 0.015)	
**Circadian rhythm syndrome**							
No	0 (Reference)		0 (Reference)	0 (Reference)		0 (Reference)	
Yes	-0.378 (-0.421, -0.335)	**	-0.019 (-0.057, 0.019)	-0.123 (-0.172, -0.073)	**	-0.032 (-0.053, -0.01)	**
