# Supplementary material for: Associations of Solid Fuel Use and Circadian Rhythm Syndrome With Physical Function and Muscle Strength in Middle-Aged and Older Adults: Nationwide Cohort Study in China
Source: JMIR Aging. 2026 Jun 29;9:e78352. doi: 10.2196/78352 (PMC13365896; doi:10.2196/78352)
Supplement: Multimedia Appendix 9 [file aging_v9i1e78352_app9.pdf]

**Variables**	**Muscle strength**		**Gait speed**	**Chair stand test**		**Balance**	
	β (95%CI)		β (95%CI)	β (95%CI)		β (95%CI)	
Household fuel use							
Clean fuel	0 (Reference)		0 (Reference)	0 (Reference)		0 (Reference)	
Solid fuel	-0.023 (-0.063, 0.016)		-0.026 (-0.063, 0.01)	-0.146 (-0.192, -0.1)	**	-0.021 (-0.04, -0.002)	*
Circadian syndrome							
No	0 (Reference)		0 (Reference)	0 (Reference)		0 (Reference)	
Yes	-0.304 (-0.342, -0.265)	**	-0.022 (-0.058, 0.014)	-0.124 (-0.169, -0.079)	**	-0.033 (-0.052, -0.015)	**
