# Supplementary material for: Associations of Solid Fuel Use and Circadian Rhythm Syndrome With Physical Function and Muscle Strength in Middle-Aged and Older Adults: Nationwide Cohort Study in China
Source: JMIR Aging. 2026 Jun 29;9:e78352. doi: 10.2196/78352 (PMC13365896; doi:10.2196/78352)
Supplement: Multimedia Appendix 10 [file aging_v9i1e78352_app10.pdf]

Type	Household fuel use	Circadian rhythm syndrome component	
		n	IRR (95% CI)
Individual effects			
	Cooking fuel use		
	Clean fuel	3107	1 (Reference)
	Solid fuel	4827	1.026 (0.998, 1.055)
	Heating fuel use		
	Clean fuel	3114	1 (Reference)
	Solid fuel	4820	1.043 (1.014, 1.073)
Combined effects			
	Cooking and heating fuel use		
	Clean fuel use for both cooking and heating	1913	1 (Reference)
	Solid fuel use for either cooking or heating	2395	1.026 (0.990, 1.064)
	Solid fuel use for both cooking and heating	3626	1.055 (1.018, 1.093)
