# Supplementary material for: Associations of Solid Fuel Use and Circadian Rhythm Syndrome With Physical Function and Muscle Strength in Middle-Aged and Older Adults: Nationwide Cohort Study in China
Source: JMIR Aging. 2026 Jun 29;9:e78352. doi: 10.2196/78352 (PMC13365896; doi:10.2196/78352)
Supplement: Multimedia Appendix 11 [file aging_v9i1e78352_app11.pdf]

**Household fuel use**	**Association between household fuel use and physical function**		**Association between household fuel use and circadian syndrome**	
	β (95%CI)		OR (95%CI)	
Clean fuel	0 (Reference)		1 (Reference)	
Coal use	-0.276 (-0.411, -0.141)	*	1.221 (1.02, 1.463)	*
Crop residue / Wood burning use	-0.198 (-0.286, -0.11)	*	1.04 (0.922, 1.172)	
