# Supplementary material for: Associations of Solid Fuel Use and Circadian Rhythm Syndrome With Physical Function and Muscle Strength in Middle-Aged and Older Adults: Nationwide Cohort Study in China
Source: JMIR Aging. 2026 Jun 29;9:e78352. doi: 10.2196/78352 (PMC13365896; doi:10.2196/78352)
Supplement: Multimedia Appendix 12 [file aging_v9i1e78352_app12.pdf]

**Household fuel use**	**Observation**	**Physical function**
	15208	β (95%CI)
Clean fuel		0 (Reference)
Solid fuel		-0.215 (-0.288, -0.143)
