# Supplementary material for: Associations of Solid Fuel Use and Circadian Rhythm Syndrome With Physical Function and Muscle Strength in Middle-Aged and Older Adults: Nationwide Cohort Study in China
Source: JMIR Aging. 2026 Jun 29;9:e78352. doi: 10.2196/78352 (PMC13365896; doi:10.2196/78352)
Supplement: Multimedia Appendix 14 [file aging_v9i1e78352_app14.pdf]

Type	Circadian rhythm syndrome		Physical function	
	OR (95% CI)		β (95%CI)	
Cooking fuel use				
Clean fuel	1.000 (Reference)		0.000 (Reference)	
Solid fuel	1.164 (1.025, 1.322)	*	-0.208 (-0.311, -0.105)	*
Heating fuel use				
Clean fuel	1.000 (Reference)		0.000 (Reference)	
Solid fuel	1.199 (1.044, 1.378)	*	-0.272 (-0.387, -0.157)	*
Clean fuel use for both cooking and heating	1.000 (Reference)		0.000 (Reference)	
Solid fuel use for either cooking or heating	1.177 (1.012, 1.402)	*	-0.210 (-0.355, -0.065)	*
Solid fuel use for both cooking and heating	1.316 (1.118, 1.549)	*	-0.350 (-0.485, -0.216)	*
No circadian rhythm syndrome			0.000 (Reference)	
Circadian rhythm syndrome			-0.454 (-0.557, -0.350)	*
