# Supplementary material for: Associations of Solid Fuel Use and Circadian Rhythm Syndrome With Physical Function and Muscle Strength in Middle-Aged and Older Adults: Nationwide Cohort Study in China
Source: JMIR Aging. 2026 Jun 29;9:e78352. doi: 10.2196/78352 (PMC13365896; doi:10.2196/78352)
Supplement: Multimedia Appendix 15 [file aging_v9i1e78352_app15.pdf]

Type	Circadian rhythm syndrome		Physical function	
	OR (95% CI)		β (95%CI)	
Cooking fuel use				
Clean fuel	1.000 (Reference)		0.000 (Reference)	
Solid fuel	1.081 (1.075, 1.09)	*	-0.219 (-0.303, -0.135)	*
No circadian rhythm syndrome			0.000 (Reference)	
Circadian rhythm syndrome			-0.484 (-0.565, -0.402)	*
