# Supplementary material for: Associations of Solid Fuel Use and Circadian Rhythm Syndrome With Physical Function and Muscle Strength in Middle-Aged and Older Adults: Nationwide Cohort Study in China
Source: JMIR Aging. 2026 Jun 29;9:e78352. doi: 10.2196/78352 (PMC13365896; doi:10.2196/78352)
Supplement: Multimedia Appendix 16 [file aging_v9i1e78352_app16.pdf]

Variables	Physical function	
	β (95% CI)	
Clean fuel & No circadian rhythm syndrome	0.000 (Reference)	
Solid fuel & No circadian rhythm syndrome	-0.154 (-0.255, -0.053)	*
Clean fuel & Circadian rhythm syndrome	-0.39 (-0.508, -0.273)	*
Solid fuel & Circadian rhythm syndrome	-0.709 (-0.826, -0.593)	*
